# Neighbourhood-level socioeconomic status and prevalence of teacher-reported health disorders among Canadian kindergarten children

**DOI:** 10.3389/fpubh.2023.1295195

**Published:** 2024-01-18

**Authors:** Magdalena Janus, Marni Brownell, Caroline Reid-Westoby, Molly Pottruff, Barry Forer, Martin Guhn, Eric Duku

**Affiliations:** ^1^Offord Centre for Child Studies, Department of Psychiatry and Behavioural Neurosciences, McMaster University, Hamilton, ON, Canada; ^2^Human Early Learning Partnership, School of Population and Public Health, University of British Columbia, Vancouver, BC, Canada; ^3^Manitoba Centre for Health Policy, Department of Community Health Sciences, University of Manitoba, Winnipeg, MB, Canada

**Keywords:** child health, prevalence, neighborhood, socioeconomic status, school readiness, early development instrument, child development, health disorders

## Abstract

**Background:**

The evidence on the association between neighborhood-level socioeconomic status (SES) and health disorders in young children is scarce. This study examined the prevalence of health disorders in Canadian kindergarten (5–6 years old) children in relation to neighborhood SES in 12/13 Canadian jurisdictions.

**Methods:**

Data on child development at school entry for an eligible 1,372,980 children out of the total population of 1,435,428 children from 2004 to 2020, collected using the Early Development Instrument (EDI), were linked with neighborhood sociodemographic data from the 2006 Canadian Census and the 2005 Taxfiler for 2,058 neighborhoods. We examined the relationship using linear regressions. Children’s HD included special needs, functional impairments limiting a child’s ability to participate in classroom activities, and diagnosed conditions.

**Results:**

The neighborhood prevalence of health disorders across Canada ranged from 1.8 to 46.6%, with a national average of 17.3%. The combined prevalence of health disorders was 16.4%, as 225,711 children were identified as having at least one health disorder. Results of an unadjusted linear regression showed a significant association between neighborhood-level SES and prevalence of health disorders (*F*(1, 2051) = 433.28, *p* < 0.001), with an *R*^2^ of 0.17. When province was added to the model, the *R*^2^ increased to 0.40 (*F*(12, 2040) = 115.26, *p* < 0.001). The association was strongest in Newfoundland & Labrador and weakest in Ontario.

**Conclusion:**

Our study demonstrated that the prevalence of health disorders among kindergarten children was higher in lower SES neighborhoods and varied by jurisdiction in Canada, which has implications for practice and resource allocation.

## 1 Introduction

Where children live matters a great deal to their health, especially for those living in low socioeconomic areas (1–3). This is reflected in the association of neighborhood-level socioeconomic status (SES) with children’s health and well-being (2, 4). As explained by Hertzman and Boyce (5), early exposures and experiences can “get under the skin” and have the potential to impact one’s future health and development. Neighborhood deprivation in the early years of life contributes to these exposures and is one of the factors associated with adverse child health and developmental outcomes (6). Neighborhood-level SES has been associated with several aspects of children’s physical and mental health (2). For instance, using data from the National Longitudinal Survey of Children and Youth in the United States (7), neighborhood-level SES was found to be inversely associated with the odds of being overweight or obese in children 5 to 17 years of age, even after controlling for individual and family demographics. Similar associations were found between neighborhoods and behavioral problems in children. In a nationally representative sample of Canadian children aged 4 to 11 years old, between-neighborhood variation accounted for approximately 7% of children’s behavioral problems, as reported by parents and teachers (7.6 and 6.6%, respectively) (8). Data from the United States indicate that neighborhood characteristics, especially those indicative of SES, are strongly associated with the prevalence of health disorders (9–11). In a study using the National Study of Children’s Health (12), unadjusted analyses showed that children who had mental, behavioral, or developmental disorders were more likely to live in poorer neighborhoods, compared to their peers without these disorders. Once family-level variables were adjusted for in the analyses, however, neighborhood characteristics were no longer significantly associated with children’s outcomes, indicating a strong association between family-and neighborhood-level SES. Evidence from studies conducted using a range of methodologies, such as randomized experiments, multilevel modeling, or longitudinal studies, concludes that neighborhoods are associated with various health outcomes, even after family-level variables are taken into account, and have small to moderate effect sizes [see (1) for a review].

A growing body of place-based research in the United States is using the Child Opportunity Index (COI), a census track-level measure of disparities and resources in areas of education, health and environment, society, and economics (13). COI consists of 18 indicators and advances the study of neighborhood impact by acknowledging that it goes beyond just poverty and involves other social determinants. Using the COI, studies have found significant associations between neighborhood resources and various aspects of child health, including physical health (14), earlier puberty (15), asthma hospitalizations (16), and pediatric care use (17). Furthermore, a systematic review of multilevel studies of the association between neighborhood-level SES and children’s health and well-being found small to moderate effects of children’s health outcomes, such as birth weight, injuries, behavioral issues, and child maltreatment (18). Put together, these studies help us further understand how neighborhood-level social determinants of health may influence specific aspects of children’s physical health and acute care and suggest that neighborhood-level interventions could have beneficial effects on children.

Research on adult health shows that area-level social determinants are associated with a broad range of health and functional needs (19–21), however, these associations tend to vary depending on the country (22) and the methodology of the research being conducted (23). Msall et al. (11) demonstrated that school-aged children in neighborhoods in Rhode Island, in the United States, characterized by high levels of unemployment, single parenthood, child poverty, and high-school dropout rates, had disproportionately high rates of disability, defined as having at least one functional impairment. Children with a *health disorder*, defined as either a medical diagnosis, an identified special health need, or a functional impairment that limits one’s ability to take part in classroom activities, experience different developmental health trajectories than children without such conditions (24). In Canada, based on teacher-reported data up to 2015, the prevalence of health disorders among kindergarten children (age 5–6 years) was approximately 15% (25), which is slightly lower than the 17–20% range reported in Australia for 4–5 years-old children in 2009 and 2015 (26). Among otherwise healthy children, approximately 27% of kindergartners lack the developmental skills to take optimal advantage of school-based education, while among children with identified special health needs at that age, this proportion rises to almost 80% (27). Having a health disorder in childhood often impacts trajectories of development throughout childhood, adolescence, and adulthood (28–30). Currently, there is little evidence on the relationship between neighborhood-level SES conditions and the overall prevalence of health disorders among young children starting school, especially at the population level and in countries other than the United States and Australia (26).

In Canada, the development of children with disabilities at school entry is associated with the SES of the neighborhood where they live, and it is the poorest in neighborhoods at the lowest end of the SES spectrum (2, 31), thus showing the same pattern as observed among typically developing children (32). Little is known, however, whether in a country with universal health care, like Canada, the prevalence of children with health disorders varies according to neighborhood SES. Examining this association is important because of the free universal health care, which results in a different social and medical care landscape than in the United States (33, 34), and should minimize the place-based variation.

It is also important to acknowledge that delivery and access to health care that is universal in principle may still be affected by a plethora of social determinants of health, both family and place-based, such as parent education or migration status, and availability of public transport, to name a few (35). Most recently, these disparities are likely being exacerbated by the impacts of the COVID-19 pandemic and climate change (e.g., (36) that could be at particular risk for inequality due to area-level factors. Among the systemic factors, government funding model has been identified as one of the most powerful (37). While some barriers to accessibility of health care are dismantled through universal funding (such as affordability), others still remain (e.g., (38)).

One of the barriers in addressing the disparities for targeted populations, such as young children, is lack of evidence on their distribution across neighborhoods and jurisdictions. Availability of data on the prevalence of children’s health disorders in relation to where they live prior to or at school entry is scarce at the population level. This has limited the ability to examine jurisdictional differences and develop evidence-based policies, even though there is jurisdictional variation in the development of children with identified disabilities (39).

Because health disorders in young children have the potential to impact their future health and well-being, it is imperative to examine broader aspects of the possible association between neighborhood-level SES and the prevalence of health disorders. Since education and healthcare are mandated at the provincial/territorial level in Canada, the prevalence of health disorders may differ across provinces and territories. Previous Canadian studies, encompassing several jurisdictions, found a positive association of SES factors with the prevalence of a specific disorder, such as obesity or developmental delays (40–42). The teacher-reported Early Development Instrument (EDI) data collected in most Canadian jurisdictions, using the same methodology and including information on persistent health concerns that impair child’s ability to learn at school, offer an unprecedented opportunity to examine the prevalence of functional health disorders at school entry in Canada.

The objective of this study was to examine the association between neighborhood-level SES, as identified by population-level data for 2,058 neighborhoods from 12 of Canada’s 13 provinces and territories (32, 43), and the prevalence of children with health disorders in different provinces/territories.

## 2 Materials and methods

### 2.1 Study design and participants

This was a cross-sectional, population-wide secondary analysis study of children attending kindergarten in publicly-funded schools across Canada between the 2003/04 and 2019/20 school years from 12 of the 13 Canadian provinces and territories. It was approved by the first author’s institutional Ethics Board.

### 2.2 Measures

#### 2.2.1 Health disorders

Health disorders were assessed using data collected with EDI (44), a 103-item, teacher-completed questionnaire that measures children’s ability to meet age-appropriate developmental expectations in kindergarten and includes child’s demographic and health status. Because the EDI is completed by teachers as part of government-funded provincial/territorial implementations, it provides a data source that is unparalleled to any other dataset, as it offers population-level information on children’s school readiness, including some health questions as they pertain to child development. The EDI was completed in the second half of the school year by kindergarten teachers for each student in their class. A child was considered as having a health disorder if they were reported to have a diagnosed health condition (based on information from a parent or health professional), if they were recognized by their teacher as having a limitation that interfered with their ability to function in the classroom (e.g., physical, learning, emotional, behavioral, speech and language, other) and/or if they received a special needs designation (yes/no). It is important to note that this classification reflects child’s health in the context of the school setting and is therefore a functional designation rather than a diagnostic one (26). The various health and developmental conditions were combined into one group because we were interested in taking a non-categorical approach to health disorders. This approach aligns with the World Health Organization’s International Classification of Functioning, Disability and Health (45) which emphasizes one’s functioning rather than their specific diagnosis. Many of the conditions included are not mutually exclusive, and, in many cases, show comorbidity. All the conditions in this broad category are recurrent and interfere in some way with a child’s ability to learn at school (school readiness).

In earlier versions of the EDI, teachers responded on a paper questionnaire, using text boxes to indicate a response. Data collection transitioned to an electronic completion and these response options changed to a drop-down menu. A record was considered valid if there were fewer than 25% of the items missing on the EDI.

The EDI database is described in the data profile paper (25). Regional data are shared with school divisions and communities on demand and used in local planning. Provincial/territorial data linked with administrative data are available in British Columbia and Manitoba through secure data repository channels (46, 47). The Offord Centre for Child Studies is a repository for Canadian and international data (25).

#### 2.2.2 Neighborhood-level SES

Information on neighborhood-level SES was retrieved from the 2005 Taxfiler database and the 2006 Canadian Census, collected through Statistics Canada. An SES index identifying 10 socioeconomic variables^1^ relevant to child development was created for 2,058 custom-defined neighborhoods across the country (32). These custom neighborhoods span the whole country and were defined using Statistics Canada’s dissemination blocks (49). Neighborhoods were created based on a minimum of 50 valid EDI records and a maximum of 400–600 valid EDI records per neighborhood (48, 50). Fifty records were used as the minimum number based on a previous EDI reliability study (51) and the maximum number of 400–600 was chosen in order to denote the sociodemographic heterogeneity in urban areas (52). A comprehensive description of the neighborhood creation process is described by Guhn and colleagues (43). The SES index was transformed into *Z*-scores, with a mean of 0 and a standard deviation of 1. A higher SES index represents higher overall neighborhood SES. The neighborhood SES index was merged with the EDI dataset using children’s postal codes with a 98.8% match rate. Analyses of the SES index constructed with the same methodology on Census data from subsequent collections revealed it was highly consistent over time, with fewer than 3% of the neighborhoods with a greater than one-index quintile category change overtime (53).

### 2.3 Data analysis

Descriptive statistics were examined for demographics of children with and without health disorders. A linear regression model was developed to determine the association between the prevalence of health disorders, neighborhood SES, and province/territory in Canada. Subsequently, linear regression models were run individually for each province/territory with enough data to examine this same association. For linear regression models run separately for each province/territory, the jurisdictions with fewer than 40 neighborhoods (Northwest Territories, Yukon, and Prince Edward Island) were excluded, leaving 9/12 jurisdictions available for this analysis.

All children who met the following criteria were included in the regression models: (1) were enrolled in kindergarten; (2) were in their current classroom for at least 1 month; (3) had a questionnaire with no more than 25% of items missing; (4) had data on whether they had a health disorder; and (5) were successfully matched to a neighborhood code and associated SES index. In addition to this, neighborhoods with fewer than 25 children were excluded from analysis to maintain the anonymity of the data (there were five neighborhoods with fewer than 25 children). All statistical analyses were conducted using the statistical software SPSS, version 28 (54).

## 3 Results

### 3.1 Sample characteristics

**Figure 1:**
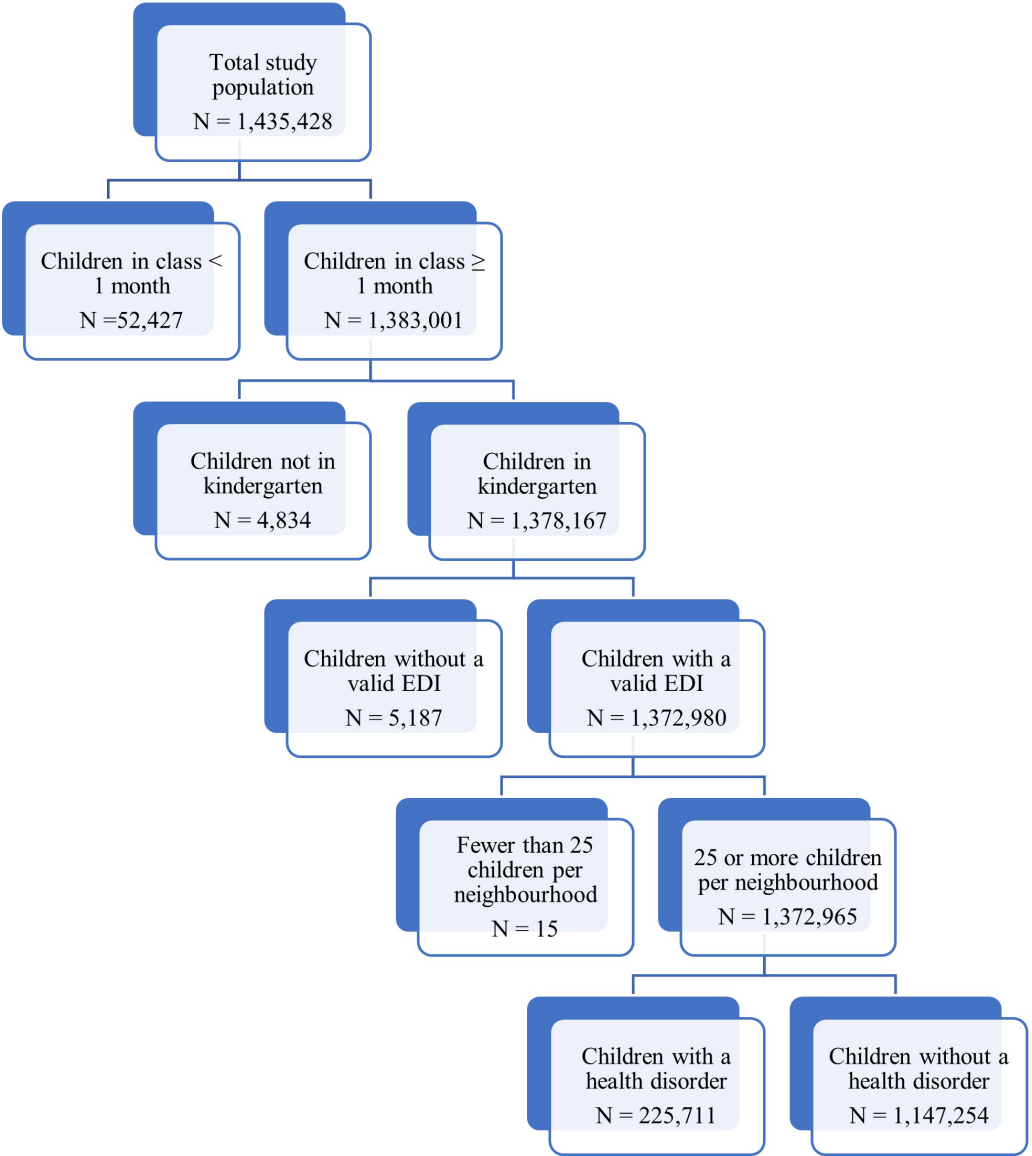
Flowchart of participants.

Of a total of 1,435,428 children who participated in the provincial/territorial EDI data collections between 2004 and 2020 in Canada, 230,021 (16.0%) had a health disorder. Figure 1 shows the flow of the number of participants in the study. After filtering out children who did not meet the inclusion criteria described above and those living in neighborhoods with fewer than 25 records, 1,372,965 children (95.6% of the total study population) remained and were therefore included in the regression analyses.

The mean age of the resulting analytic sample was 5.72 years; 51.3% were males, and 13.9% spoke English or French as a second language. In the full analytic sample, 225,711 children (16.4%) were identified as having a health disorder. Table 1 shows the breakdown of the prevalence of health disorders in each jurisdiction by year.

**Table 1:** Canadian Early Development Instrument (EDI) implementation schedule from 2004 to 2020 with percentage of children in each data collection year with a health disorder, by province/territory.

	AB	BC	MB	NB	NL	NT	NS	ON	PEI	QC	SK	Y
2004								**12.4%**				
2005	17.7%	**14.6%**	12.9%	9.0%				**14.5%**		14.2%	14.0%	
2006		**13.1%**	**18.5%**	15.9%			15.1%	**12.3%**		13.8%	14.9%	
2007		**12.1%**	**12.0%**	10.2%				**8.5%**		18.8%	14.2%	
2008	12.6%	**17.4%**	12.6%		10.9%		16.9%	**10.5%**	**8.1%**	20.1%	16.1%	
2009	**19.6%**	**13.1%**	**11.9%**	**12.1%**			12.5%	**10.9%**		14.6%	**19.0%**	
2010	**19.5%**	**19.5%**					19.7%	**15.6%**			17.1%	**9.0%**
2011	**22.9%**	**19.2%**	**17.0%**		14.3%		20.0%	**14.8%**			20.0%	**15.9%**
2012	**20.0%**	**20.1%**			15.7%	**24.3%**	20.4%	**16.5%**		**19.6%**	16.2%	**24.6%**
2013	**18.6%**	**19.3%**	**16.1%**		**14.5%**	**23.3%**	**18.9%**				18.6%	**23.8%**
2014		**17.4%**			**16.3%**	**23.8%**	22.8%					
2015		**15.6%**	**16.0%**			**26.2%**	**18.6%**	**16.8%**				
2016	22.0%	**15.0%**				**34.3%**	20.0%					
2017		**16.4%**	**17.4%**			**27.9%**				20.5%		
2018		**16.8%**				**24.0%**	20.6%	**17.4%**				
2019		**16.2%**	**16.3%**		**18.5%**	**25.6%**			**22.9%**			
2020						**24.7%**	**18.3%**					

Bold font in cells indicates a full provincial collection; if the cells are shaded, that indicates a collection spanned multiple years meaning a province or territory completed the implementation in waves. Regular font in cells indicate a partial provincial collection. AB, Alberta; BC, British Columbia; MB, Manitoba; NB, New Brunswick; NL, Newfoundland and Labrador; NT, Northwest Territories; NS, Nova Scotia; ON, Ontario; PEI, Prince Edward Island; QC, Quebec; SK, Saskatchewan; Y, Yukon.

Table 2 displays the number and percentages of children with health disorders by province/territory, across all years, for the full study population. Northwest Territories and Yukon had the highest rates of children with health disorders, while New Brunswick had the lowest proportion.

**Table 2:** Numbers and percentages of children with health disorders, by province/territory between 2004 and 2020, as well as the number of neighborhoods by province/territory.

Province	Number of neighborhoods	Number of children with health disorders (%)	Total number of children
Alberta	266	21,902 (20.7%)	128,862
British Columbia	298	40,547 (16.4%)	252,727
Manitoba	75	16,910 (15.5%)	114,582
New Brunswick	52	1,074 (12.1%)	9,192
Newfoundland and Labrador	41	2,841 (16.0%)	18,167
Northwest Territories	3	1,342 (26.0%)	5,662
Nova Scotia	57	8,574 (18.4%)	48,239
Ontario	798	92,568 (14.8%)	646,495
Prince Edward Island	6	421 (16.1%)	2,649
Quebec	396	32,381 (19.5%)	166,816
Saskatchewan	55	6,793 (17.7%)	40,562
Yukon	6	360 (25.1%)	1,475
Total	2,053	225,711 (16.4%)	1,372,965

Among children with health disorders, there was a higher percentage of males (65.9% vs. 48.4%, *χ*^2^ (1, *N* = 1,372,965) = 23233.86, *p* < 0.001) and a lower percentage of children who spoke English or French as their second language (13.5% vs. 14.0%, *χ*^2^ (1, *N* = 1,372,965) = 40.84, *p* < 0.001), compared to their peers without health disorders (Table 3). Children with health disorders were similar in age to their peers without health disorders but lived in neighborhoods with a lower average SES (*z*-score −0.13 vs. 0.04, all *p* < 0.001).

**Table 3:** Description of included children with and without health disorders.

Variables	Children with health disorders	Children without health disorders
	Number (%)	Number (%)
Males	148,844 (65.9%)	555,285 (48.4%)
English/French as a second language	30,531 (13.5%)	161,034 (14.0%)

	Mean (SD)	Mean (SD)
Mean age (SD)	5.72 (0.35)	5.72 (0.32)
Mean neighborhood-level SES (z-score)	−0.13 (0.99)	0.04 (1.01)

SD, standard deviation; SES, socioeconomic status.

### 3.2 Prevalence of health disorders by neighborhood SES

**Figure 2:**
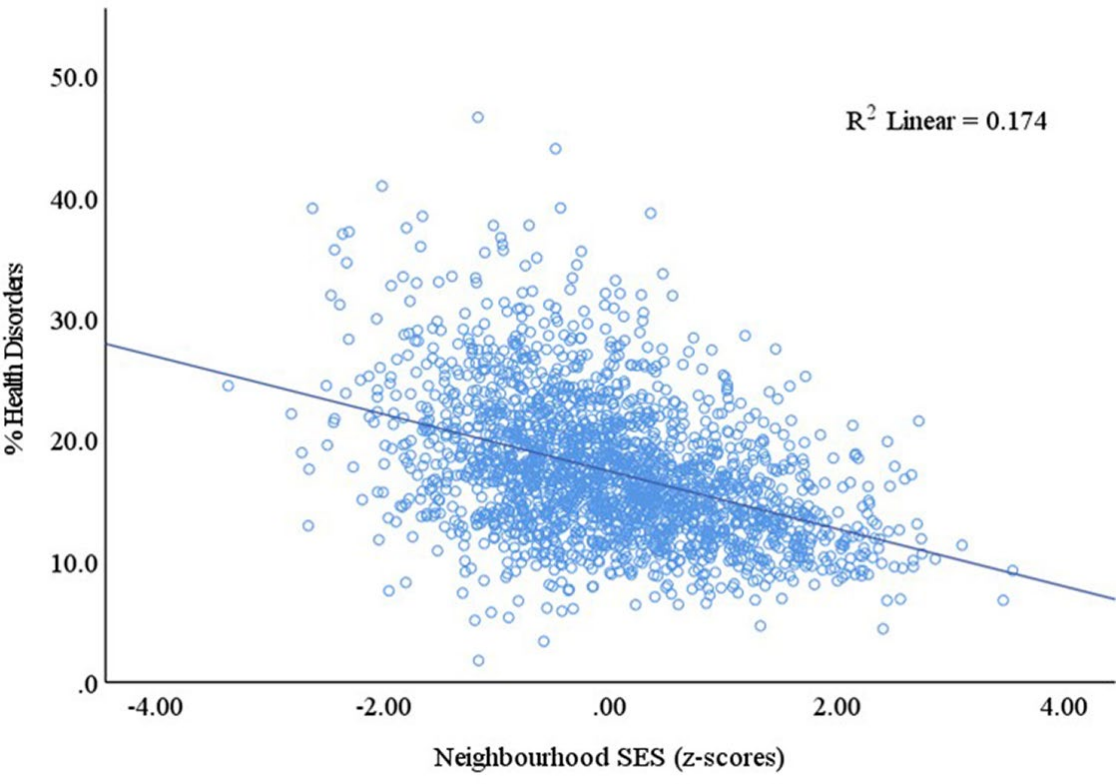
Linear association between the prevalence of health disorders in kindergarten children and neighborhood-level SES in Canada.

The prevalence of health disorders in all Canadian neighborhoods ranged from 1.8 to 46.6%, (mean = 17.3%, SD = 5.66). Unadjusted linear regression revealed a significant association between neighborhood-level SES and prevalence of health disorders (*F*(1, 2051) = 433.28, *p* < 0.001), with an *R*^2^ of 0.17 (Figure 2). For one standard deviation decrease in neighborhood-level SES, the prevalence of health disorders increased by 2.37%. A scatterplot of standardized predicted values compared to standardized residuals demonstrated that the data met the assumptions of homogeneity of variance, as well as linearity. The residuals were also normally distributed.

When jurisdiction of the neighborhood (province/territory) was added to the model, the *R*^2^ increased to 0.40 (*F*(12, 2040) = 115.26, *p* < 0.001). For one standard deviation decrease in neighborhood-level SES, the prevalence of health disorders increased by 2.45%. Separate regressions for nine jurisdictions with adequate numbers of neighborhoods showed that the strength of the association between neighborhood SES and the prevalence of health disorders was highest in Newfoundland & Labrador and weakest in Quebec (Table 4). There was no significant association between the prevalence of health disorders and neighborhood SES in New Brunswick.

**Table 4:** Neighborhood prevalence of health disorders and its association with neighborhood-level SES, by jurisdiction.

Jurisdiction	Range of prevalence of HD by neighborhoods	Coefficients (95% CI)	*p*
Alberta	6.8–46.6%	3.58 (3.00–4.17)	<0.001
British Columbia	5.9–36.7%	3.04 (2.52–3.55)	<0.001
Manitoba	6.9–37.7%	2.39 (1.50–3.28)	<0.001
New Brunswick	1.8–25.5%	1.25 (−0.46–2.96)	0.149
Newfoundland and Labrador	7.8–32.9%	4.63 (2.23–7.03)	<0.001
Northwest Territories	23.9–28.2%	Insufficient number of neighborhoods	
Nova Scotia	9.3–26.8%	4.27 (2.91–5.62)	<0.001
Ontario	4.4–39.1%	2.07 (1.81–2.32)	<0.001
Prince Edward Island	10.5–19.6%	Insufficient number of neighborhoods	
Quebec	8.9–37.4%	1.96 (1.43–2.47)	<0.001
Saskatchewan	10.1–38.7%	2.14 (0.40–3.88)	0.017
Yukon	18.3–32.1%	Insufficient number of neighborhoods	
Total	1.8–46.6%	2.37 (2.15–2.59)	<0.001

SES, socioeconomic status; HD, health disorders; CI, confidence intervals.

## 4 Discussion

The goal of this population-level study was to establish the level of the association between the prevalence of health disorders in kindergarten children and the SES of the neighborhood in which they live in 12/13 Canadian jurisdictions. Findings indicated that Canadian children living in poorer neighborhoods were more likely to have health disorders at school entry, with the strength of that association varying by jurisdiction. A previous study showed that Canadian kindergarten children with disabilities were proportionally more likely to also have poorer developmental health the lower the SES of neighborhoods they lived in (39). Combined, these results indicate that young children with compromised health experience increased odds of being exposed to factors that may set them at a disadvantageous developmental trajectory.

Our study is in line with previous research that has found that, in high-income countries, childhood disorders are associated with social disadvantage (55). For instance, a negative association was previously found between the prevalence of chronic childhood disabilities and SES in the United States (56), and research from Australia demonstrated that children living in disadvantaged neighborhoods had higher odds of having a special health care need (26). Growing up in lower SES neighborhoods suggests an overall health disadvantage for children, which, in turn, has been suggested to set individuals on disadvantageous health and development trajectories (57–59). These variations could be attributable, at least in part, to the availability and funding of programs (60), or geographic disparities in the distribution of healthcare practitioners and services.

Not only did the prevalence of health disorders at school entry vary by neighborhood, with lower SES neighborhoods having a greater likelihood of having higher rates of children with health disorders, our study demonstrated that the strength of this association varied by province/territory. The association between the prevalence of health disorders and neighborhood SES was strongest in Newfoundland and Labrador and weakest in New Brunswick (not significant). There are several possible reasons for this. For one, it is possible the SES gradient is steeper in some provinces/ territories than in others (2). Furthermore, income inequality, that is, the extent to which income is unevenly distributed in a given area (61), appears to differ by jurisdiction. Based on Canadian-wide data from 2015 to 2020, Prince Edward Island and New Brunswick had the smallest after-tax income inequality and Alberta had the largest (62), which might explain the strength of the association we found. In Canada, there seems to be a general trend towards greater income equality as one moves from west to east (61).

We found that there was a slightly higher percentage of children who did not speak one of Canada’s official languages (English or French) among those without teacher-reported health disorders than those with (14% vs. 13.5%), which was unexpected. This is one of the subpopulations in our study that is worth further investigating in future research, especially with datasets that allow integration of family-level information on children’s immigration status and their health.

Our observed provincial/territorial differences could also be due to varying policies between provinces. More specifically, policies about the schooling of children with special needs vary by province and territory, and even across regions and school districts within a given province (63, 64). Many policies, such as those surrounding the educational and health systems, are mandated by each province/territory, leading to differences in how education and health systems are administered across the country. Some differences in policy include disparities in the criteria employed to establish which children are eligible to receive services, the types of services provided to children with similar difficulties, the allocation of resources for offering these services, and the use of special education classes (64).

Our findings infer important implications for policy and practice. Knowing that the association between the prevalence of health disorders in kindergarten and neighborhood-level SES is stronger in some areas of the country than others can help us identify opportunities to support children with health disorders in these areas and reduce the level of variability across provinces, improving the outcomes for children with health disorders. Even though universal health care system exists in Canada, our findings point to the growing potential and importance of direct income policies and supports (e.g., national child benefit tax credits) that can raise the incomes of families in lower SES neighborhoods as well as early childhood development and education programs that can prevent, delay, or treat health disorders. Additionally, our results suggest that communities with lower SES than those more affluent should have a greater and more equitable provision of public health goods (e.g., initiatives for nutrition, housing, access to quality health services and preventive care) to mitigate health disadvantages. As provinces have some freedom to decide their budgetary allocations for the health and education sector, the health spending expenditures may also have an impact on the prevalence of health disorders in kindergarten children. Using data from the Canadian Community Health Survey from 2007/08 and 2015/16, Lavergne and colleagues (38) noted that many variables, such as income, education, dwelling ownership, immigration, racialization, and sex/gender, were associated with disparities in access to primary care, despite the legislated universality.

### 4.1 Strengths and limitations

Our study had several strengths, such as the population-level coverage and the sample size of over 1.3 million children. Our study had data for kindergarten children across the entire country, with the exception of one territory, making it the most comprehensive study of health disorder prevalence in young children in Canada. Because of our population-wide design, using teachers as respondents, and a broad approach in defining health disorders, we achieved a comprehensive coverage and considerable number of children with health disorders in our study [upwards of 90% coverage of all children attending kindergarten in publicly-funded schools in Canada (65)], allowing us to examine the association of prevalence with neighborhood-level SES. Future research should examine the associations found in the current study while also considering distance and access to services. Also, the use of a non-categorical approach, by describing children as having health disorders rather than grouping based on specific diagnoses, was also advantageous. Approaches that rely on diagnostic categories have been previously disputed and criticized for their failure to capture the varying degrees of impairment or the complexity and overlapping of conditions, and the inability to reflect the actual abilities of children (66, 67). Our definition of health disorders was more inclusive by focusing on functioning in the school setting and recognized the intricacy of children’s disabilities and impairments. It also allowed us to increase our numbers, enabling us to examine the relationship between prevalence and neighborhood-level SES in less populated areas of the country.

However, we recognize that our health disorder category represented varying types and degrees of impairments and disabilities, which resulted in a very heterogeneous group. A broad approach to the operationalization of health disorders was intentional since complete diagnostic information is seldom available for children in kindergarten as many are just starting the process of medical evaluation. Because of the small number of kindergarten children with any given diagnosis in a given school year, schools are unable to tailor interventions to specific conditions. The lack of health-professional confirmation of children’s disorders or their severity is another limitation of our study. We were also unable to account for potential confounders of the association between the prevalence of health disorders and neighborhood-level SES such as the distribution of healthcare practitioners and services and type of practice (68). While the collection of data spanning 16 years is a strength, it can also be a limitation, as regulations for classification of special needs, for example, could have shifted over time. Finally, the mode of questionnaire completion changed over time. In the earlier versions of the EDI, teachers responded on a paper questionnaire, using text boxes. As data collection moved to an electronic completion, these response options changed to a drop-down menu. It is possible that differences in response options could have impacted the data slightly, e.g., by making it easier to record the information.

Despite the limitations, this study is an important first step in investigating the prevalence of health disorders across Canada and its association with neighborhood-level SES. Future research should aim to use administrative databases with more in-depth data on specific health diagnoses, despite the potential limitation of much smaller sample size, as administrative health data in Canada are so far mostly available only for one jurisdiction at a time, and in some, not at all.

## 5 Conclusion

Our population-level study demonstrated that (1) a sizeable number of children are identified by their teachers as having a health disorder of some kind, (2) the prevalence of health disorders is negatively associated with area-level SES, and (3) the strength of this association varies by jurisdiction. While associations with area-level SES have been found for adult health, the results of our national-level study emphasize the SES-related inequality in child health and development – children presenting to school with health disorders that require additional support disproportionately live in lower-SES neighborhoods. As our study included data up to spring 2020, it may also serve as a baseline for future assessment of children’s health disorders since the beginning of the COVID-19 pandemic. Policymakers and researchers alike may need to focus more on these children to ensure they are properly supported, especially in school, as this is an important opportunity to help improve their long-term outcomes.

## Data availability statement

Publicly available datasets were analyzed in this study. This data can be found at: Instructions provided in the Resource paper: https://ijpds.org/article/view/431.

## Ethics statement

This study was approved by the Hamilton Integrated Research Ethics Board (HiREB). The study was conducted in accordance with the local legislation and institutional requirements. Written informed consent for participation was not required from the participants or the participants’ legal guardians/next of kin in accordance with the national legislation and institutional requirements.

## Author contributions

MJ: Conceptualization, Funding acquisition, Writing – original draft, Writing – review & editing. MB: Conceptualization, Funding acquisition, Writing – review & editing. CR-W: Formal analysis, Writing – original draft, Writing – review & editing. MP: Data curation, Formal analysis, Project administration, Writing – review & editing. BF: Conceptualization, Methodology, Writing – review & editing. MG: Conceptualization, Methodology, Writing – review & editing. ED: Conceptualization, Methodology, Writing – review & editing.

## Footnotes

^1^The 10 variables used to develop the SES index were: percentage with low income, lone parent families with children under 6; percentage separated or divorced individuals; percentage with incomes twice or higher than the provincial median, families with children under 6; percentage with union/association dues, families with children under 6; percentage with investment income, families with children under 6; percentage non-migrant movers in the past year; percentage with charitable donations, families with children under 6; percentage with no high school diploma; percentage individuals not speaking either official language at home; Gini Coefficient quintile, lone female families with children under 6 (48).
